# Is Alcohol Consumption Associated with Poor Perceived Academic Performance? Survey of Undergraduates in Finland

**DOI:** 10.3390/ijerph17041369

**Published:** 2020-02-20

**Authors:** Walid El Ansari, Abdul Salam, Sakari Suominen

**Affiliations:** 1 Department of Surgery, Hamad General Hospital, Doha 3050, Qatar; 2 College of Medicine, Qatar University, Doha 3050, Qatar; 3 School of Health and Education, University of Skovde, 541 28 Skövde, Sweden; sakari.suominen@his.se; 4 Neuroscience Institute, Hamad General Hospital, Doha 3050, Qatar; abdul.or.salam@gmail.com

**Keywords:** heavy episodic drinking, problem drinking, alcohol dependence, university students, sociodemographic and educational characteristics

## Abstract

The relationship between academic performance and alcohol consumption among students remains inconsistent. We assessed this relationship, controlling for sociodemographic characteristics across seven faculties at the University of Turku (1177 undergraduates). An online questionnaire assessed: seven sociodemographic characteristics (age, gender, year/discipline of study, accommodation type, being in intimate relationship, parental education, and income sufficiency); two perceived academic performance (students’ subjective importance of achieving good grades and students’ appraisal of their academic performance compared to peers); and six alcohol consumption behaviors (length of time, amount consumed, frequency, heavy episodic drinking, problem drinking, and possible alcohol dependence). Simple logistic regression assessed relationships between sociodemographic and academic variables with alcohol consumption behaviors; multiple logistic regression assessed the same relationships after controlling for all other variables. Students reported long duration and large amount of drinking (46% and 50%), high frequency of drinking (41%), heavy episodic drinking (66%), problem drinking (29%), and possible alcohol dependence (9%). After controlling, gender was associated with all alcohol consumption behaviors, followed by religiosity (associated with four alcohol behaviors), living situation, marital status, age (each associated with two alcohol behaviors), and parental education and year of study (each associated with one alcohol behavior). Study discipline, income sufficiency, importance of achieving good grades, and academic performance compared to peers were not associated with any alcohol behaviors. Universities need to assess problem drinking and alcohol use disorders among students. Prevention strategies are required to reduce risk. Health promotion efforts could focus on beliefs and expectations about alcohol and target student groups at risk for more efficient and successful efforts.

## 1. Introduction

Alcohol contributes to over 200 diseases and injury-related health conditions [1,2]. Hence, inappropriate alcohol use among college students is a major global public health concern, due to its multiple and wide ranging direct or indirect effects on physical, psychosocial, and mental health. For instance, heavy episodic drinking (HED, aka binge drinking), characterized by the consumption of large amounts of alcohol in a short time, followed by a period of abstinence, is particularly common among young people, especially university students [3,4]. In Spain, there is a high (20–30%) prevalence of HED among young adults [5], and this HED level is within the average of the European Union [6]. Likewise, across seven universities in England, Wales, and Northern Ireland (3706 students), 37.9% reported long duration of drinking, 46.5% reported large amount of drinking, and 42.7% exhibited high frequency of drinking [7]. Among college students, in Tunis, the prevalence of risky alcohol consumption and alcoholic disorder were 52.5% and 79.1% respectively [8]; prevalence of frequent binge drinking was 13.8% in France, [9]; prevalence of alcohol use was 35.5% in Ethiopia [10]; and, in the USA, college-age adults had a high prevalence of alcohol use disorders [11].

A body of research evidences the relationship between alcohol consumption, and the deterioration of cognitive functions and worsening academic performance. For example, excessive alcohol consumption is associated with disorders of memory, attention, and planning [12,13,14,15,16,17]; daily drinking was significantly negatively associated with academic performance [18]; and problematic alcohol use among university students was associated with poor academic performance [19]. The relationship between HED and academic engagement, performance, and future aspirations among secondary school students suggests that students who initiate binge drinking have poor school performance and engagement, which may hinder achieving their future academic goals [20]. HED students are more likely to miss class, fall behind in their schoolwork, and perform poorly on a test(s) or academic project(s) [21]. Likewise, heavy drinking among youth is linked to lower school grades [22,23,24,25], truancy [22,26,27], and degree noncompletion [28]. High alcohol consumption levels are associated with poor academic performance among young university students [29].

Many sociodemographic variables play a confounding role in the relationship between alcohol consumption and academic performance among young adults. On the one hand, alcohol use is related to sociodemographic features, e.g., gender [9,30,31], place of residence or living at the family home [6,9,32,33,34,35], financial situation or disposable income [31,33,34,36], paternal education [37], and having an intimate relationship [38]. However, many inconsistencies exist. For instance, among younger adults, studies show inconsistent relationships between alcohol intake and social position [38,39,40,41]; and research on alcohol use and academic performance reported no relationship, a negative relationship or a positive relationship [19,42,43]. On the other hand, academic performance is related to sociodemographic variables, e.g., gender, parents’ (maternal and paternal) education [44,45], religion [46], age [45,47], discipline of study [45], income sufficiency and monthly allowance [45,48], and living circumstances (accommodation) during the university semester [7].

Very few studies assessed alcohol consumption behaviors and/or academic performance among university students in Finland. Segregated studies examined some variables in isolation, younger or older age groups, or did not include academic performance, [49,50,51]; included one type of alcohol consumption behavior among medical students only [52]; or were outdated [53]. This is despite that the levels of alcohol consumption in Finland, although close to the OECD average, have increased over the last 30 years [54]; and the prevalence of HED in 2016 among those aged 15–19 years in the population was 31.9% and among drinkers only of the same age was 51.6% [55]. To the best of our knowledge, no previous study investigated alcohol use among young adult populations of university students in Finland, mobilizing a range of alcohol consumption behaviors and controlling for a range of sociodemographic variables.

Therefore, the current study at the University of Turku in Finland assessed the associations between students’ (a) sociodemographic characteristics (age, gender, year/discipline of study, accommodation, intimate relationship, parental education, and income sufficiency), and (b) academic performance characteristics (importance of good grades, academic performance relative to peers) on the one hand; and (c) six alcohol consumption behaviors (length of time of the last drinking occasion, amount of alcohol consumed during the last drinking occasion, high frequency of drinking, frequency of HED, problem drinking, and possible alcohol dependence) on the other. The study objectives were to describe the prevalence of alcohol consumption behaviors, and to assess whether sociodemographic characteristics and academic performance were associated with alcohol consumption behaviors. The study also evaluated the alcohol consumption behavior(s) that were mostly associated with academic performance, controlling for sociodemographic characteristics.

## 2. Materials and Methods

### 2.1. Ethics, Sample, and Data Collection

An initial ‘invitation to participate’ emails were sent to all undergraduates at all faculties at the University defining the aims and objectives of the study and encouraging students to go online and complete the survey. Participation was voluntary and anonymous (no academic or monetary incentives were provided), and data were confidential and protected at all times. Students were informed that by completing the online survey, they agreed to participate in the study. Two weeks after the initial email invitation of students, a follow up reminder email was sent again to all undergraduates. In addition, three posters about the study were displayed at the students’ cafeteria at the University, and a reminder was posted on the University intraweb. A pilot survey was undertaken first (May 2013, random sample, 200 students) stratified by faculties. Very few participants reported any comprehensibility challenges related to the English questionnaire, and the number of missing values related to items that reasonably could be expected to be answered by all respondents was trivial. The main survey was then launched with the unmodified questionnaire (September 2013). The pilot sample was excluded from the final eligible sample that included 4387 undergraduates at the University of Turku. The Research and Ethics Committee at the University approved the study (Approval # Lausunto 10/2010), and survey data were collected using a secure online self-administered English questionnaire (2013–2014) at the University of Turku in Turku, Finland.

As students completed the online survey and clicked the ‘submit’ button, their responses were saved and directed to the Student Management Office at the University. The Student Management Office gathered the completed online responses, and the data were electronically entered into an excel sheet ensuring high quality assurance. After this phase was completed, the data was sent to the research team who electronically imported the data (no identifiers) into SPSS for the analysis. The total number of responses received was 1177. Students’ mean age was about ≈23 (SD 5) years and 832 (70.4%) were females. Based on the number of returned questionnaires, the response rates were about 27%. 

### 2.2. Health and Wellbeing Questionnaire

The Health and Wellbeing Questionnaire employed in student health and wellbeing surveys. The tool was used and field-tested among university student populations across many countries [7,38,42,48,56,57,58,59]. The questionnaire included sociodemographic information (e.g., sex age, marital status, parental education, accommodation during university terms, income sufficiency, and religiosity, i.e., importance of religious faith), academic related questions (e.g., year and discipline of study at university, importance of achieving good grades, and academic performance compared to peers), as well as self-reported health behavior data (e.g., five alcohol consumption behaviors, HED, problem drinking, and possible alcohol dependence).

#### 2.2.1. Sociodemographic Variables

Age, gender, year, and discipline of study at university was self-reported by the students.

Living arrangements (accommodation during university semesters/terms; 1 item): “Where do you live during university/college term time?” dichotomized into ‘living with parents’ vs. ‘not living with parents’.

Socioeconomic status (SES; 2 items): “What is the highest education level of your father?” The same question was also asked about mother’s education level (no formal education, primary school, secondary school, high school, bachelor’s degree, master’s degree and Ph.D. or equivalent). For the current analysis, we employed the highest education of either parent.

Income sufficiency (1 item): “How sufficient do you consider your income?” with four Likert scale responses (“always sufficient”, “mostly sufficient”, “mostly insufficient” or “insufficient”) which were then dichotomized into “always sufficient” vs. “other”. 

Religiosity (personal importance of religious faith): the extent to which participants agreed/disagreed with the statement: “My religion is very important for my life”, 1 = ‘strongly agree’, 2 = ‘somewhat agree’, 3 = ‘neither agree nor disagree’, 4 = ‘somewhat disagree’, and 5 = ‘strongly disagree’, later recoded into two categories based on agreement/ disagreement (1, 2, 3 = 1 vs. 4, 5 = 2).

Discipline of study: students were asked about the faculty they were enrolled at in the University of Turku, and discipline they were studying. For the current analysis we collapsed the seven faculties into five.

#### 2.2.2. Alcohol Consumption Variables (5 Items)

Length of time of the last (most recent) drinking occasion (1 item): “The last time you ‘partied’/socialized, how many hours did you drink alcohol?” Participants provided the number of hours. As the median and mean were almost the same (mean = 4.37, median = 4 h), we used a mean split of the number of hours and dichotomized this variable into a ‘High’ vs. ‘Low’ length of time of drinking.

Amount (number of drinks) of alcohol consumed during the last (most recent) drinking occasion (1 item): “The last time you ‘partied’/socialized, how many alcoholic drinks did you have? (including alcoholic drinks you possibly had before going out)”. Participants provided the number of drinks. A “drink” is defined a glass of wine (ca 15 cl), a bottle or can of beer (ca 50 cl), a shot glass of spirits (ca 5 cl), or a mixed drink. As the median and mean were almost the same (mean = 5.54, median = 5 drinks), in line with other research [7], we used a mean split of the number of drinks and dichotomized this variable into a ‘High’ vs. ‘Low’ amount of drinking. 

Frequency of alcohol consumption (1 item): “Over the past 3 months how often did you drink alcohol, for example, beer?” (six response options: “never”, “once a week or less”, “once a week”, “a few times each week”, “every day”, and “a few times each day”), later dichotomized into Low frequency = “drinking once a week or less” vs. High frequency = “drinking a few times or more each week”. 

Heavy episodic drinking (1 item): “Think back again over the last 30 days. How many times (if any) have you had five or more drinks on one occasion?” (A “drink” is a glass/bottle/can of beer (≈50 cl), a glass/bottle/can of cider (≈50 cl), two glasses/bottles of alcopops (≈50 cl), a glass of wine (≈15 cl), a glass of spirits (≈5 cl), or a mixed drink). Response options were “never”, “once”, “twice”, “3–5 times”, “6–9 times”, and “10 or more times”. Respondents were classified into non-heavy episodic drinkers (if they responded “never”) vs. heavy episodic drinkers (all others).

Problem drinking (4 items): An alcoholism-screening Cut-down, Annoyed, Guilty, Eye-opener (CAGE) test [60] comprising of 4 questions (Have you ever felt you should cut down on your drinking? Have people annoyed you by criticizing your drinking? Have you ever-felt bad or guilty about your drinking? Have you ever had a drink in the morning to get rid of a hangover? (eye opener). Each question is answered either “yes” or “no”. Two or more affirmative answers suggested problem drinking. We categorized respondents as non-problem (<2 positive responses) vs. problem drinkers (≥2 positive responses). 

Possible alcohol dependence (4 items): ≥3 positive CAGE responses [60] can suggest alcohol dependence. We categorized respondents as not possible alcohol dependence (<3 positive responses) vs. possible alcohol dependence (≥3 positive responses).

#### 2.2.3. Educational (Academic Performance) Variables (2 Items)

Academic performance was measured in the questionnaire by using two indicators [48]:

Students’ internal reflection on their academic performance (importance they attach to achieving good grades): “How important is it for you to have good grades at university?” (4 response categories, 1 = ‘very important’, 2 = ‘somewhat important’, 3 = ‘not very important’, and 4 = ‘not at all important’). We dichotomized this variable into 1 = ‘very important’ vs. 2 = ‘other’. 

Students’ subjective comparative appraisal of their overall academic performance (in comparison with their peers) “How do you rate your performance in comparison with your fellow students?” (5 response categories, 1 = ‘much better’, 2 = ‘better’, 3 = ‘same’, 4 = ‘worse’, and 5 = ‘much worse’). We dichotomized this variable into 1 = ‘Same, better or much better’ vs. 2 = ‘worse or much worse’.

### 2.3. Statistical Analysis

Descriptive and inferential statistics were used to characterize the study sample and test hypotheses. Descriptive results for all quantitative variables (e.g., age) are presented as mean ± standard deviation (SD; for normally distributed data). Numbers (percentage) were reported for all qualitative variables (e.g., gender). Binomial distribution measured the prevalence of the 6 alcohol consumption behaviors. The relationship between socio demographic and educational characteristics and each of the 6 alcohol consumption behaviors was assessed by an independent sample t-test, Pearson chi-square test, or Fisher exact test, as appropriate. In terms of predictors, a simple logistic regression assessed the relationship between socio demographic and academic characteristics with each of the six alcohol consumption behaviors (crude odds ratio and 95% confidence interval for the odds ratio). In order to account for any potential effects of the other variables under examination, multiple logistic regression analysis assessed the relationship between academic performance and alcohol consumption, controlling for sociodemographic characteristics (adjusted odds ratio and 95% CI for adjusted odds ratio). *p* value < 0.05 (two-tailed) was considered statistically significant. Statistical analyses were performed using Statistical Package for Social Sciences Version 22 (SPSS).

## 3. Results

### 3.1. Participating Faculties and Disciplines

A wide range of faculties and disciplines of the University of Turku participated in the current study. Participating students were enrolled at all the seven faculties of the University of Turku (Humanities, Mathematics and Natural Sciences, Medicine, Law, Social Sciences, Education, and Economics). Students represented a large variety of disciplines of study, including adult education, special education, pedagogy, languages, philosophy, law, accounting, finance, economics, marketing, medicine, nursing, dentistry, psychology, biomedicine, health bioscience, archaeology, biochemistry, biology, chemistry, mathematics, geography, history, political science, social science, computer science, information technology, and biotechnology.

### 3.2. General Characteristics of the Sample

Selected characteristics of the study population are depicted in Table 1. Females were more represented across the sample, and about one third of participants lived with their parents during university terms. About half the students were either married or in a relationship, and nearly one third had both parents with a high educational level. Slightly more than half the sample (58%) had a disposable income that was mostly or always sufficient. Over 50% of respondents somewhat or strongly disagreed that religion was important in their life. Nearly half the respondents were first year students, and in terms of discipline, over a quarter of sample studied Technology and various Sciences, another quarter were Humanities students, while each of the Education and Law, Economics, and Medicine disciplines roughly comprised of 12–16% of the sample. Most respondents reported that achieving good grades was somewhat or very important for them, and rated their own academic performance compared to their peers as the same, better, or much better. Roughly 41–50% of participants reported a long duration of drinking, a large amount of drinking, or a high frequency of drinking alcohol; slightly >25% were problem drinkers, while a minority (≈9%) of respondents exhibited possible alcohol dependence.

### 3.3. Distribution of Students’ Characteristics by Six Alcohol Consumption Behaviors

Table 2 depicts the distribution of students’ characteristics by alcohol consumption behaviors. Males and students in the later years of studies (≥4th year) were more represented across all alcohol consumption behaviors. Students of Education and Law had a slightly higher percentage of possible alcohol dependence. Living with parents and being single displayed mostly lower odds across five alcohol behaviors. ‘Mostly or always sufficient’ income sufficiency and those who somewhat or strongly agreed that religion was important in their lives both displayed lower odds across the alcohol consumption behaviors. Respondents for whom achieving good grades was somewhat or very important, and felt that their academic performance compared to their peers was the same, better, or much better displayed lower odds across the alcohol consumption behaviors. 

### 3.4. Predictors of Six Alcohol Consumption Behaviors (before Controlling for Effects of Other Variables)

Table 3 shows the simple logistic regression analysis of sociodemographic and academic performance predictors of alcohol consumption behaviors before controlling. Increasing age was protective against HED but conversely, positively associated with possible alcohol dependence. Males were more likely to engage in all alcohol consumption behaviors, particularly large amount of drinking and possible alcohol dependence. Year and discipline of study were both not significantly related to any alcohol consumption behaviors. Students living with their parents were less likely to engage in large amounts of drinking. Married students or those in an intimate relationship were more likely to report possible alcohol dependence. Low parental education protected against high frequency of drinking. Students with ‘mostly or always insufficient’ disposable income were more likely to engage in long duration and large amount of drinking, and possibly be alcohol dependent. Lower levels of religiosity were associated with higher likelihood of engagement in four alcohol behaviors (long duration, large amount, and high frequency of drinking, HED). Students for whom achieving good grades was important, or those who rated their academic performance compared to peers as ‘same, better, or much better’ were generally less likely to engage in all alcohol consumption behaviors, but none of these relationships reached statistical significance. In addition, we undertook this simple logistic regression analysis separately for males and females (data not presented), and the findings did not change much.

### 3.5. Predictors of Six Alcohol Consumption Behaviors (after Controlling for Effects of Other Variables)

Table 4 shows the multiple logistic regression analysis of sociodemographic and academic predictors of six alcohol consumption behaviors after controlling. Increasing age was protective against HED but conversely, positively associated with high frequency of drinking. Males were more likely to engage in all alcohol consumption behaviors. Lower years of study were protective against problem drinking. Discipline of study was not associated with alcohol behaviors. Students living with parents during university terms were less likely to engage in large amounts of drinking and problem drinking. Married students or those in an intimate relationship were more likely to report a large amount of drinking and possible alcohol dependence. Lower parental education protected against high frequency of drinking. Perceived income sufficiency was not associated with any alcohol behaviors. Lower levels of religiosity were associated with higher likelihood of engagement in four alcohol behaviors (long duration, large amount, and high frequency of drinking, HED). Students for whom achieving good grades was important, or those rating their academic performance compared to peers as ‘same, better, or much better’ were generally less likely to engage in all alcohol consumption behaviors, but none of these relationships reached statistical significance.

### 3.6. Which Predictors Are Associated with Many Aalcohol Consumption Behaviors?

Table 5 summarizes the association(s) of each variable with the number and types of alcohol consumption behaviors, before and after controlling. After controlling, gender was the most pronounced variable, associated with all alcohol behaviors, followed by religiosity (associated with four behaviors), and living situation, marital status, and age (each associated with two behaviors). Parental education and year of study were each associated with one alcohol behavior. The remaining three variables (discipline of study, perceived income sufficiency, importance of achieving good grades, and academic performance compared to peers) were not associated with any alcohol behaviors.

## 4. Discussion

HED is the most common form of alcohol misuse in adolescents and young adults [61], and the 66% rate of the current study supports the literature. For instance, among students at seven universities in England, Wales, and Northern Ireland, using the same questionnaire as the current study, ≈59–70% of students reported HED at least once within the last 2 weeks [7]. Similar to our findings, 67.2% of Slovakian undergraduates reported HED [38]. Likewise, 47.6% of students in Spain were binge drinkers [14], and 61% of students in England were positive for Alcohol Use Disorder Identification Test (AUDIT) scores [62]. Acute negative alcohol-related consequences have a dose–response relationship with binge drinking [63], where more frequent binge episodes are linked to higher risk of adverse consequences [64,65,66]. Among young adults, after accounting for the overall quantity of alcohol consumed, binging adversely influenced brain functioning [67], effects that could, with time, impact on academic performance. As brain maturation continues into young adulthood, it is unclear whether the effects of HED on brain structure and functioning are reversible with persistent abstinence [68,69].

In terms of problem drinkers, our 29% level agreed with the 13.5–29% problem drinker students in the United Kingdom [7]. Others similarly reported a 16–27% prevalence of problem drinking among university students from Denmark, Germany, Spain, Lithuania, Poland, Bulgaria, and Turkey [70]. In Finland, 33% of medical students at one university were risky drinkers [52]; and in Ethiopia, using the valid and reliable Alcohol Use Disorder Identification Test (AUDIT) [71], 11.4% of the sample were problematic alcohol users of which 6.8% and 4.6% exhibited medium and high level problems respectively [18]. In agreement, across all universities in Finland, 33.4% of students had AUDIT score ≥8 (hazardous, harmful, or alcohol-dependent use of alcohol) [72]. The use of different instruments to measure alcohol consumption across different studies hinders the precise and direct comparisons between countries. Comparisons of our findings with others were challenging, due to the diversity of approaches of measurement of alcohol use/misuse (e.g., time period of recall; whether the number of drinks or actual alcohol units were employed in estimating the amount of alcohol consumed, cut-offs used for calculating HED, etc.). Hence, we agree with others about the need for agreement on alcohol screening tools and their cut-offs to permit comparisons between studies [73].

For alcohol dependence, our 9% possible alcohol dependence is within other reported levels among students, e.g., 5.2–11.4% (United Kingdom) [7], 9% ‘probably alcohol dependent’ (England) [64], and, 10% probable alcohol dependence (developed countries) [62,74]. Although students in six European countries had a mean 3% probable dependence, those from Northern European countries had significantly higher AUDIT scores compared with Central and Southern European students [73]. The current study was in northern Europe, and students with possible alcohol abuse are a concern to educators, universities, and the health services, particularly that our use of self-administered survey might underestimate the prevalence of problematic alcohol use and probable alcohol dependence. Combining our problem drinkers (29%) and those with possible dependence (9%), more than one in three (38%) of these students could have an alcohol use disorder. Research would benefit from investigating the beliefs/behavior of students who fall in these alcohol consumption categories.

As for the relationships between academic performance and alcohol, evidence supports an inverse relationship between alcohol use among university students and academic performance [18,21,42,75]. Among our sample, the academic performance variables were both not associated with alcohol consumption behaviors. This is in partial support of research in the United Kingdom that used the same instrument and reported that the importance of achieving good grades was not associated with alcohol consumption behaviors, while the ‘same or better’ academic performance compared to peers was significantly protective against problem drinking and possible alcohol dependence [7]. 

Whilst our lack of a relationship between the academic performance variables and the alcohol consumption variables contrasts with other studies, it nevertheless supports longitudinal studies that reported paradoxical effects between alcohol use and education [24,76]. For instance, research found either no or a positive relationship between alcohol use and academic performance. In the USA, alcohol use frequency before enrollment was positively associated with the odds of graduate degree completion (adjusted odds ratio = 1.007) [43]. Students who drink might be more likely to engage in the academic environment and stimulate social support [77], particularly as there were prospective relationships between alcohol use and higher subjective well-being and increased self-efficacy [78,79] among students. Compared to sober peers, students consuming moderate to high levels of alcohol and low marijuana demonstrate lower GPAs, but this difference became non-significant over time [71,80]. Likewise, the association between alcohol use and GPA disappeared when controlling was undertaken for other factors [81,82] akin to the control of sociodemographic variables we undertook. Such findings emphasize the complicated relationships between alcohol consumption and academic performance [43], and we agreed with others [43] in that such findings should not propose that drinking is either associated with or does not influence academic performance. In addition, analysis of the literature is impeded by the range of measures of academic performance employed and the use of degree completion as an outcome measure [20]. School dropout is possibly preceded by a period of student apathy, deteriorating grades, truancy, and/or other problems [20].

In terms of sociodemographics, we found that increasing age was protective against HED, but conversely, positively associated with high frequency of drinking, in partial agreement with Nigeria [37]. In the current study, males were positively associated with two alcohol behaviors, supporting findings in the USA and United Kingdom [62,83]. We also found that students living with parents were less likely to engage in large amounts of drinking, in agreement with that heavy drinking was more prevalent when living on campus [84]. Others similarly reported that accommodation with parents during the semester was negatively associated with high frequency of drinking, heavy episodic drinking, and problem drinking [7]. Our observation that parental education was associated with one alcohol behavior supports other research, where adolescent drinking was associated with adolescents’ own but not with parental socioeconomic position [49]. Others found no relationships between alcohol consumption variables and various combinations of parental educational [7]. Parental education seems selective in terms of the alcohol behaviors it is associated with; in Slovakia, higher parental education was associated with lower levels of problem drinking, but not with frequency of alcohol use, frequency of drunkenness, and HED [38]. The same indicators of socioeconomic status should be used in research on socioeconomic differences in health behavior so that findings of different studies are comparable [85].

Our findings also indicate that perceived income sufficiency was not associated with alcohol behaviors, in contrast with the United Kingdom, where perceived income insufficiency was significantly associated with all alcohol behaviors examined [7]. Others [86] found significant relationships between monthly income and being ever substance user but undertook only bivariate analysis, not controlling for a range of factors. Finally, our finding that lower levels of religiosity were associated with higher likelihood of engagement in four alcohol behaviors agrees with that religiosity was negatively associated with frequency of drinking and HED among both genders [56]. Religiosity is a factor associated with the protection of individuals against alcohol consumption [87,88,89].

A final point pertains to the changes that we observed in the relationships between the various variables after controlling was undertaken. The changes in number, types, strength (magnitude), and significance level of the relationships highly suggest that controlling for the relevant variables should be mandatory in such studies in order to try to unpack the complex and intricate relationships between the variables that collectively interplay in the relationships between alcohol consumption and other sociodemographic variables and academic performance.

This study had limitations and generalizations require caution. The study was cross-sectional, so relationships were associations not causations. All data was self-reported (potential recall bias social desirability and sociability cannot be ruled out). We are unable to conclude whether self-reports underestimate alcohol consumption [90], or whether students might overestimate consumption levels in self-reports [91]. Objective measures of drinking (e.g., breath alcohol concentration) [92], or objective assessment of academic performance (e.g., actual module grades or GPA) would have been beneficial. Neither the university nor the students were selected at random: participants were recruited at one university, and despite reminder emails, this sample remains a convenience sample. Non-response could be due to problem drinking, and students less/uninterested in healthy practices might be less motivated to partake and hence were under-represented. The survey was administered only at one point in time, however, abrupt changes can occur in the alcohol consumption trajectories of young adults [93]. We had no data on academic performance before enrolment at university, and this might have influenced respondents’ drinking patterns at university. We observed no relationships between alcohol consumption behaviors and academic performance (non-significant associations were present). This could be due to the actual lack of association, or due methodological issues, e.g., sample size and statistical power of the study might explain some of the null associations [94]. Nevertheless, it is acknowledged that it is difficult to predict students’ grades generally, whether by using sociodemographic and academic/educational variables [95]. Future research should attempt to address these limitations.

Despite these limitations, the study has important strengths. To the best of our knowledge, no previous study, particularly in Finland, seems to have investigated the detailed prevalence and predictors of six different alcohol consumption behaviors and, the associations between such use and two academic performance variables across a sample of students from many different faculties. Likewise, we consciously incorporated two different academic outcomes to help elucidate the relationships between drinking on the one hand, and two ‘proximal’ indicators of academic performance, namely students’ internal reflection on their academic performance as well as students’ subjective comparative appraisal of their overall academic performance on the other hand. This is rather than focusing only on ‘distal’ indicators of academic performance, e.g., degree attainment or GPA that could overlook any adverse influences of drinking on academic performance and engagement among students who manage to graduate [20]. 

## 5. Conclusions

After controlling, gender was associated with all alcohol behaviors, followed by religiosity (associated with four behaviors), living situation, marital status, age (each associated with two behaviors), and parental education and year of study (each associated with one behavior). Study discipline, income sufficiency, importance of achieving good grades, and academic performance compared to peers were not associated with any alcohol behaviors. Universities need to assess problem drinking and alcohol use disorders among students. Prevention strategies are required to reduce risk. Health promotion efforts could focus on beliefs and expectations about alcohol and target student groups at risk for more efficient and successful efforts.

## Figures and Tables

**Table 1:** Students’ sociodemographic, academic, and alcohol consumption characteristics.

Variable	Results *N* (%)
Sociodemographic	
Age (years) M ± SD	22.95 ± 5.21
Gender	
Male	346 (29.6)
Female	823 (70.4)
Living during university terms	
With parents	394 (33.7)
Other accommodation	776 (66.3)
Marital status	
Married or in relationship	593 (50.7)
Single	576 (49.3)
Parental educational level	
Both parents low	137 (19.8)
Mother low, father high	133 (19.2)
Mother high, father low	174 (25.1)
Both parents high	249 (35.9)
Perceived income sufficiency	
Mostly or always insufficient	488 (42.0)
Mostly or always sufficient	674 (58.0)
Religiosity	
Somewhat or strongly disagree	702 (60.2)
Neither agree nor disagree	238 (20.4)
Somewhat or strongly agree	226 (19.4)
Academic	
Years of study	
1st year	553 (47.2)
2nd year	344 (29.4)
3rd year	251 (21.4)
≥4th year	23 (2.0)
Discipline of Study at University	
Education and Law	188 (16.4)
Economics	138 (12.0)
Medicine	168 (14.6)
Technology and Science	328 (28.5)
Humanities	327 (28.5)
Importance of achieving good grades	
Somewhat or very important	971 (83.1)
Not important or at all important	198 (16.9)
Academic performance compared to peers	
Same, better or much better	992 (84.6)
Worse or much worse	180 (15.4)
Alcohol consumption	
Long duration of drinking ^a^	542 (49.5)
Large amount of drinking ^b^	503 (45.6)
High frequency of drinking ^c^	480 (41.0)
Heavy episodic drinking ^d^	729 (66.2)
Problem drinkers ^e^	329 (28.8)
Possible alcohol dependence ^f^	100 (8.7)

^a^ >4.37 mean hours during last (most recent) drinking occasion; ^b^ >5.54 mean number of drinks during last (most recent) drinking occasion; ^c^ drinking a few times or more each week over past 3 months; ^d^ ≥5 alcoholic drinks at a sitting during last 30 days; ^e^ ≥ 2 positive Cut-down, Annoyed, Guilty, Eye-opener (CAGE) responses; ^f^ ≥3 positive CAGE responses.

**Table 2:** Students’ sociodemographic and academic characteristics by six alcohol consumption behaviors.

	Long Duration of Drinking ^a^ *N* (%)	Large Amount of Drinking ^b^ *N* (%)	High Frequency of Drinking ^c^ *N* (%)	Heavy Episodic Drinking ^d^ *N* (%)	Problem Drinking ^e^ *N* (%)	Possible Alcohol Dependence ^f^ *N* (%)
Gender						
Female	361 (47.2)	309 (39.9)	311 (38.0)	490 (63.7)	215 (26.7)	58 (7.2)
Male	178 (55.1)	192 (59.4)	167 (48.3)	236 (72.4)	112 (33.5)	41 (12.3)
Year of study at university						
1st year	256 (49.8)	240 (46.4)	222 (40.2)	337 (65.4)	143 (26.7)	40 (7.5)
2nd year	152 (47.1)	147 (45.1)	148 (43.3)	218 (66.9)	95 (28.0)	32 (9.4)
3rd year	121 (52.4)	103 (44.0)	99 (39.8)	158 (67.5)	80 (32.9)	25 (10.3)
≥4th year	13 (59.1)	13 (59.1)	11 (47.8)	15 (68.2)	10 (43.5)	3 (13.0)
Discipline of Study at University						
Education and Law	83 (48.3)	82 (47.1)	77 (41.0)	114 (65.1)	54 (29.7)	22 (12.1)
Economics	65 (51.2)	58 (45.3)	57 (41.9)	90 (71.4)	43 (32.1)	9 (6.7)
Medicine	86 (53.4)	79 (49.1)	66 (39.5)	110 (69.2)	46 (27.7)	14 (8.4)
Technology and Science	145 (47.5)	129 (41.9)	124 (38.0)	189 (61.2)	88 (27.6)	23 (7.2)
Humanities	156 (51.5)	148 (48.4)	141 (43.3)	207 (67.9)	89 (28.3)	29 (9.2)
Living during university terms						
With parents	179 (47.5)	153 (40.4)	147 (37.5)	242(64.0)	102 (26.3)	38 (9.8)
Other accommodation	361 (50.6)	348 (48.3)	330 (42.7)	486(67.6)	225 (30.0)	62 (8.3)
Marital status						
Married or in relationship	293 (52.0)	263 (46.1)	230 (39.0)	381 (67.2)	169 (29.0)	64 (11.0)
Single	248 (47.1)	240 (45.4)	248 (43.1)	345 (65.1)	157 (28.2)	36 (6.5)
Parental education						
Both parents low	59 (46.8)	51 (40.8)	45 (32.8)	80 (62.5)	36 (27.7)	13 (10.0)
Mother low, father high	59 (49.6)	52 (43.3)	55 (41.4)	82 (68.3)	40 (31.5)	11 (8.7)
Mother high, father low	85 (51.5)	80 (47.3)	75 (43.6)	112 (67.9)	53 (30.8)	16 (9.3)
Both parents high	112 (47.1)	115 (48.1)	110 (44.4)	165 (69.6)	61 (25.2)	16 (6.6)
Perceived income sufficiency						
Mostly or always insufficient	251 (54.3)	229 (49.5)	210 (43.1)	313 (68.2)	149 (31.0)	53 (11.0)
Mostly or always sufficient	287 (46.4)	270 (43.1)	267 (39.8)	409 (65.0)	178 (27.4)	47 (7.2)
Religiosity						
Somewhat or strongly disagree	354 (52.8)	332 (49.2)	308 (44.1)	462 (69.2)	199 (28.9)	62 (9.0)
Neither agree nor disagree	117 (53.2)	101 (45.5)	98 (41.2)	153 (68.0)	62 (26.6)	21 (9.0)
Somewhat or strongly agree	69 (35.2)	68 (34.3)	72 (32.0)	112 (56.0)	66 (31.0)	16 (7.5)
Importance of achieving good grades						
Somewhat or very important	445 (48.9)	408 (44.4)	397 (41.1)	594 (65.3)	274 (29.0)	82 (8.7)
Not important or at all important	94 (52.5)	91 (50.8)	81 (41.1)	129 (69.4)	53 (27.5)	17 (8.8)
Academic performance compared to peers						
Same, better or much better	454 (48.9)	417 (44.5)	397 (40.2)	610 (65.4)	269 (27.8)	83 (8.6)
Worse or much worse	87 (53.4)	85 (51.8)	83 (46.1)	118 (71.5)	60 (34.7)	17 (9.8)

^a^ >4.37 mean hours during last (most recent) drinking occasion; ^b^ >5.54 mean number of drinks during last (most recent) drinking occasion; ^c^ once a week to several times per day over past 3 months; ^d^ ≥5 alcoholic drinks at a sitting during last 30 days; ^e^ ≥2 positive CAGE responses; ^f^ ≥3 positive CAGE responses.

**Table 3:** Simple logistic regression: Sociodemographic and academic predictors of six alcohol consumption behaviors.

	Long Duration of Drinking ^a^ COR (95% CI)	Large Amount of Drinking ^b^ COR (95% CI)	High Frequency of Drinking ^c^ COR (95% CI)	Heavy Episodic Drinking ^d^ COR (95% CI)	ProblemDrinking ^e^ COR (95% CI)	Possible Alcohol Dependence ^f^ COR (95% CI)
Age (years)	1.0 (0.97–1.2)	0.98 (0.95–1.00)	1.01 (0.98–1.03)	0.97 (0.95–0.99) *	1.01 (0.98–1.03)	1.05 (1.02–1.08) **
Gender						
Male	1.37 (1.05–1.78) *	2.20 (1.69–2.87) ***	1.52 (1.18–1.96) ***	1.49 (1.12–1.98) **	1.38 (1.04–1.82) *	1.80 (1.18–2.74) **
Female	1 (ref)	1 (ref)	1 (ref)	1 (ref)	1 (ref)	1 (ref)
Year of study at university						
1st year	0.68 (0.28–1.63)	0.60 (0.25–1.42)	0.73 (0.31–1.69)	0.88 (0.35–2.20)	0.47 (0.20–1.10)	0.53 (0.15–1.89)
2nd year	0.61 (0.25–1.48)	0.56 (0.23–1.36)	0.83 (0.35–1.93)	0.94 (0.37–2.37)	0.50 (0.21–1.19)	0.69 (0.19–2.46)
3rd year	0.76 (0.31–1.85)	0.54 (0.22–1.32)	0.72 (0.30–1.69)	0.97 (0.37–2.47)	0.63 (0.26–1.51)	0.76 (0.21–2.75)
≥4th year	1 (ref)	1 (ref)	1 (ref)	1 (ref)	1 (ref)	1 (ref)
Discipline of study at university						
Education and Law	0.87 (0.60–1.27)	0.95 (0.65–1.38)	0.91 (0.63–1.31)	0.88 (0.59–1.31)	1.07 (0.71–1.60)	1.35 (0.75–2.43)
Economics	0.98 (0.65–1.49)	0.88 (0.58–1.33)	0.94 (0.63–1.41)	1.18 (0.75–1.86)	1.20 (0.77–1.85)	0.71 (0.32–1.54)
Medicine	1.08 (0.73–1.58)	1.02 (0.70–1.50)	0.85 (0.58–1.25)	1.06 (0.70–1.60)	0.97 (0.64–1.48)	0.90 (0.46–1.77)
Technology and Science	0.85 (0.62–1.17)	0.76 (0.55–1.05)	0.80 (0.58–1.10)	0.74 (0.53–1.03)	0.96 (0.68–1.36)	0.76 (0.43–1.35)
Humanities	1 (ref)	1 (ref)	1 (ref)	1 (ref)	1 (ref)	1 (ref)
Living situation during university terms						
With parents	0.88 (0.68–1.13)	0.72 (0.56–0.93) *	0.80 (0.62–1.03)	0.85 (0.65–1.10)	0.83 (0.63–1.09)	1.20 (0.79–1.84)
Other accommodation	1 (ref)	1 (ref)	1 (ref)	1 (ref)	1 (ref)	1 (ref)
Marital status						
Married or in relationship	1.22 (0.96–1.54)	1.03 (0.81–1.30)	0.84 (0.66–1.06)	1.09 (0.85–1.41)	1.04 (0.80–1.34)	1.78 (1.16–2.73) **
Single	1 (ref)	1 (ref)	1 (ref)	1 (ref)	1 (ref)	1 (ref)
Parental education						
Both parents low	0.99 (0.64–1.52)	0.74 (0.48–1.15)	0.61 (0.39–0.94)*	0.72 (0.46–1.14)	1.13 (0.70–1.83)	1.56 (0.73–3.37)
Mother low, father high	1.11 (0.71–1.71)	0.82 (0.53–1.28)	0.88 (0.57–1.35)	0.94 (0.58–1.51)	1.36 (0.85–2.19)	1.33 (0.60–2.98)
Mother high, father low	1.19 (0.80–1.77)	0.96 (0.65–1.43)	0.97 (0.65–1.43)	0.92 (0.58–1.41)	1.32 (0.85–2.04)	1.44 (0.70–2.98)
Both parents high	1 (ref)	1 (ref)	1 (ref)	1 (ref)	1 (ref)	1 (ref)
Perceived income sufficiency						
Mostly or always insufficient	1.37 (1.08–1.75) **	1.29 (1.01–1.64) *	1.14 (0.90–1.45)	1.15 (0.89–1.41)	1.19 (0.91–1.54)	1.58 (1.05–2.39) *
Mostly or always sufficient	1 (ref)	1 (ref)	1 (ref)	1 (ref)	1 (ref)	1 (ref)
Religiosity						
Somewhat or strongly disagree	2.06 (1.48–2.86) ***	1.85 (1.33–2.57) ***	1.67 (1.22–2.30) ***	1.76 (1.27–2.43) **	0.90 (0.64–1.26)	1.21 (0.68–2.15)
Neither agree nor disagree	2.09 (1.40–3.10) ***	1.59 (1.07–2.36) **	1.48 (1.01–2.17) *	1.67 (1.12–2.48) *	0.80 (0.53–1.21)	1.22 (0.61–2.40)
Somewhat or strongly agree	1 (ref)	1 (ref)	1 (ref)	1 (ref)	1 (ref)	1 (ref)
Importance of achieving good grades						
Somewhat or very important	0.86 (0.62–1.19)	0.77 (0.56–1.06)	0.99 (0.73–1.36)	0.83 (0.59–1.17)	1.07 (0.76–1.52)	0.98 (0.56–1.70)
Not important or at all important	1 (ref)	1 (ref)	1 (ref)	1 (ref)	1 (ref)	1 (ref)
Academic performance compared to peers						
Same, better or much better	0.83 (0.59–1.16)	0.74 (0.53–1.03)	0.78 (0.57–1.08)	0.75 (0.52–1.08)	0.72 (0.51–1.02)	0.86 (0.49–1.49)
Worse or much worse	1 (ref)	1 (ref)	1 (ref)	1 (ref)	1 (ref)	1 (ref)

COR: crude odds ratio, analysis not adjusted for any of the variables in the table; CI: confidence interval; ^a^ >4.37 mean hours during last (most recent) drinking occasion; ^b^ >5.54 mean number of drinks during last (most recent) drinking occasion; ^c^ once a week to several times per day over past 3 months; ^d^ ≥5 alcoholic drinks at a sitting during last 30 days; ^e^ ≥2 positive CAGE responses; ^f^ ≥3 positive CAGE responses; * significant at p < 0.05; ** significant at *p* < 0.01; *** significant at *p* < 0.001

**Table 4:** Multiple logistic regression: Sociodemographic and academic predictors of six alcohol consumption behaviors.

	Long Duration of Drinking ^a^ AOR (95% CI)	Large Amount of Drinking ^b^ AOR (95% CI)	High Frequency of Drinking ^c^ AOR (95% CI)	Heavy Episodic Drinking ^d^ AOR (95% CI)	Problem Drinking ^e^ AOR (95% CI)	Possible Alcohol Dependence ^f^ AOR (95% CI)
Age (years)	0.98 (0.94–1.02)	0.95 (0.91–1.00)	1.04 (1.00–1.08) *	0.93 (0.90–0.97) **	0.99 (0.95–1.03)	1.01 (0.95–1.07)
Gender						
Male	1.48 (1.02–2.16) *	2.31 (1.58–3.39) ***	1.80 (1.25–2.59) **	1.85 (1.22–2.82) **	1.48 (1.00–2.20) *	2.22 (1.20–4.09) *
Female	1 (ref)	1 (ref)	1 (ref)	1 (ref)	1 (ref)	1 (ref)
Year of study at university						
1st year	0.69 (0.24–1.97)	0.50 (0.17–1.47)	0.75 (0.27_2.03)	1.15 (0.39–3.36)	0.29 (0.11–0.80) *	0.38 (0.09–1.58)
2nd year	0.46 (0.16–1.35)	0.42 (0.14–1.25)	0.87 (0.31–2.41)	1.06 (0.36–3.16)	0.28 (0.10–0.79) *	0.47 (0.11–1.98)
3rd year	0.60 (0.20–1.74)	0.45 (0.15–1.35)	0.62 (0.22–1.73)	1.14 (0.38–3.43)	0.33 (0.12–0.93) *	0.46 (0.10–2.00)
≥4th year	1 (ref)	1 (ref)	1 (ref)	1 (ref)	1 (ref)	1 (ref)
Discipline of study at university						
Education and Law	0.87 (0.52–1.47)	0.78 (0.46–1.33)	0.70 (0.42–1.17)	0.79 (0.45–1.38)	0.81 (0.47–1.41)	0.83 (0.35–1.94)
Economics	1.30 (0.74–2.29)	0.97 (0.55–1.71)	1.08 (0.62–1.87)	1.30 (0.69–2.46)	0.81 (0.44–1.49)	0.69 (0.24–1.98)
Medicine	1.21 (0.64–1.94)	0.92 (0.52–1.61)	1.14 (0.66–1.95)	1.01 (0.55–1.83)	0.82 (0.45–1.48)	0.79 (0.31–2.03)
Technology and Science	1.05 (0.68–1.63)	0.94 (0.60–1.46)	0.92 (0.60–1.41)	0.85 (0.53–1.35)	0.84 (0.52–1.34)	0.65 (0.30–1.44)
Humanities	1 (ref)	1 (ref)	1 (ref)	1 (ref)	1 (ref)	1 (ref)
Living situation during university terms						
With parents	0.78 (0.52–1.19)	0.52 (0.34–0.79) **	0.74 (0.48–1.13)	0.89 (0.56–1.41)	0.61 (0.38–0.97) *	0.56 (0.27–1.15)
Other accommodation	1 (ref)	1 (ref)	1 (ref)	1 (ref)	1 (ref)	1 (ref)
Marital status						
Married or in relationship	1.43 (0.95–2.16)	1.77 (1.16–2.70) **	0.93 (0.62–1.39)	1.40 (0.89–2.19)	1.19 (0.77–1.84)	2.42 (1.19–4.92) *
Single	1 (ref)	1 (ref)	1 (ref)	1 (ref)	1 (ref)	1 (ref)
Parental education						
Both parents low	0.94 (0.58–1.51)	0.77 (0.47–1.25)	0.54 (0.34–0.87) *	0.79 (0.48–1.32)	1.25 (0.74–2.11)	1.62 (0.69–3.79)
Mother low, father high	1.16 (0.72–1.85)	0.82 (0.51–1.33)	0.84 (0.53–1.32)	0.95 (0.57–1.59)	1.46 (0.88–2.42)	1.57 (0.67–3.66)
Mother high, father low	1.11 (0.72–1.70)	0.91 (0.59–1.41)	0.98 (0.64–1.49)	0.84 (0.53–1.34)	1.50 (0.94–2.40)	1.55 (0.70–3.40)
Both parents high	1 (ref)	1 (ref)	1 (ref)	1 (ref)	1 (ref)	1 (ref)
Perceived income sufficiency						
Mostly or always insufficient	1.33 (0.95–1.86)	1.29 (0.91–1.82)	1.34 (0.95–1.87)	1.15 (0.79–1.66)	1.08 (0.75–1.56)	1.13 (0.62–2.06)
Mostly or always sufficient	1 (ref)	1 (ref)	1 (ref)	1 (ref)	1 (ref)	1 (ref)
Religiosity						
Somewhat or strongly disagree	2.38 (1.53–3.70) ***	2.47 (1.57–3.89) ***	1.96 (1.28–3.00) **	2.35 (1.52–3.62) ***	0.95 (0.60–1.50)	1.28 (0.58–2.79)
Neither agree nor disagree	2.84 (1.64–4.90) ***	2.06 (1.18–3.59) *	1.89 (1.12–3.21) *	2.64 (1.50–4.64) **	1.02 (0.58–1.80)	1.14 (0.42–3.06)
Somewhat or strongly agree	1 (ref)	1 (ref)	1 (ref)	1 (ref)	1 (ref)	1 (ref)
Importance of achieving good grades						
Somewhat or very important	0.98 (0.61–1.57)	0.89 (0.55–1.45)	0.76 (0.48–1.20)	0.85 (0.50–1.43)	0.84 (0.51–1.37)	1.16 (0.48–2.77)
Not important or at all important	1 (ref)	1 (ref)	1 (ref)	1 (ref)	1 (ref)	1 (ref)
Academic performance compared to peers						
Same, better or much better	0.73 (0.45–1.18)	0.69 (0.42–1.13)	0.75 (0.47–1.18)	0.64 (0.37–1.13)	0.79 (0.48–1.31)	0.79 (0.36–1.78)
Worse or much worse	1 (ref)	1 (ref)	1 (ref)	1 (ref)	1 (ref)	1 (ref)

AOD: Adjusted odds ratio, analysis adjusted for all variables in the table; CI: confidence interval; ^a^ > 4.37 mean hours during last (most recent) drinking occasion; ^b^ >5.54 mean number of drinks during last (most recent) drinking occasion; ^c^ once a week to several times per day over past 3 months; ^d^ ≥5 alcoholic drinks at a sitting during last 30 days; ^e^ ≥2 positive CAGE responses; ^f^ ≥3 positive CAGE responses; * significant at *p* < 0.05; ** significant at *p* < 0.01; *** significant at *p* < 0.001.

**Table 5:** Importance of variables to alcohol consumption behaviors: Summary of findings before and after controlling.

Variable	Alcohol Consumption Behaviors Significantly Associated with Variable				Strength and Direction of Association between Behavior and Variable ^c^
	Before Controlling ^a^		After Controlling ^b^		
	Number of Behaviors	Which Behavior(s)?	Number of Behaviors	Which Behavior(s)?	
Gender	6	All 6 behaviors	6	All 6 behaviors	Males 1.4–2.31 times more likely depending on behavior
Religiosity	4	Long duration of drinking	4	Long duration of drinking	Lower religiosity 1.89–2.84 times more likely depending on degree of religiosity and on behavior
		Large amount of drinking		Large amount of drinking	
		High frequency of drinking		High frequency of drinking	
		Heavy episodic drinking		Heavy episodic drinking	
Perceived income sufficiency	3	Long duration of drinking	0	None of the behaviors	No Association
		Large amount of drinking			
		Possible dependence			
Age	2	Heavy episodic drinking	2	Heavy episodic drinking	Older 0.93 times less likely heavy episodic drinking
		Possible alcohol dependence		High frequency of drinking	Older 1.04 times more likely high frequency of drinking
Living situation during university terms	1	Large amount of drinking	2	Large amount of drinking Problem drinking	Living with parents 0.52 and 0.61 times less likely large amount of drinking and problem drinking respectively
Marital status	1	Possible alcohol dependence	2	Large amount of drinking Possible alcohol dependence	Married or in relationship 1.77 and 2.42 times more likely large amount of drinking and possible alcohol dependence respectively
Parental education	1	High frequency of drinking	1	High frequency of drinking	Both parents low education 0.54 times less likely high frequency of drinking
Year of study at university	0	None of the behaviors	1	Problem drinking	Lower year of study 0.28–0.33 times less likely problem drinking depending on year
Discipline of study at university	0	None of the behaviors	0	None of the behaviors	No Association
Importance of achieving good grades	0	None of the behaviors	0	None of the behaviors	No Association
Academic performance compared to peers	0	None of the behaviors	0	None of the behaviors	No Association

^a^ No controlling for other variables under examination (crude odds ratios, simple logistic regression); ^b^ controlling for other variables under examination (adjusted odds ratios and multiple logistic regression); ^c^ when controlling was undertaken for the other variables under examination, i.e., based on adjusted odds ratios.
